# An Endophytic *Pseudonocardia* Species Induces the Production of Artemisinin in *Artemisia annua*

**DOI:** 10.1371/journal.pone.0051410

**Published:** 2012-12-12

**Authors:** Jie Li, Guo-Zhen Zhao, Ajit Varma, Sheng Qin, Zhi Xiong, Hai-Yu Huang, Wen-Yong Zhu, Li-Xing Zhao, Li-Hua Xu, Si Zhang, Wen-Jun Li

**Affiliations:** 1 Key Laboratory of Microbial Diversity in Southwest China, Ministry of Education and Laboratory for Conservation and Utilization of Bio-Resources, Yunnan Institute of Microbiology, Yunnan University, Kunming, China; 2 Key Laboratory of Marine Bioresource Sustainable Utilization CAS, RNAM Center for Marine Microbiology, Guangdong Key Laboratory of Marine Materia Medica, South China Sea Institute of Oceanology, Chinese Academy of Sciences, Guangzhou, China; 3 Amity Institute of Microbial Technology, Amity University Uttar Pradesh, Noida, India; 4 Key Laboratory of Biotechnology for Medicinal Plant of Jiangsu Province, Jiangsu Normal University, Xuzhou, China; 5 School of Forestry Resources, Southwest Forestry University, Kunming, China; 6 Key Laboratory of Biogeography and Bioresource in Arid Land CAS, Xinjiang Institute of Ecology and Geography, Chinese Academy of Sciences, Ürümqi, China; Institut Pasteur Paris, France

## Abstract

Endophytic actinobacteria colonize internal tissues of their host plants and are considered as a rich and reliable source of diverse species and functional microorganisms. In this study, endophytic actinobacterial strain YIM 63111 was isolated from surface-sterilized tissue of the medicinal plant *Artemisia annua*. We identified strain YIM 63111 as a member of the genus *Pseudonocardia*. *A*. *annua* seedlings grown under both sterile and greenhouse conditions were inoculated with strain YIM 63111. The growth of *A. annua* seedlings was strongly reduced when YIM 63111 was inoculated at higher concentrations under sterile conditions. However, no growth inhibition was observed when *A. annua* was grown under greenhouse conditions. Using an enhanced *green fluorescent protein* (EGFP) expressing YIM 63111 strain, we also observed the endophytic colonization of *A. annua* seedling using confocal laser-scanning microscopy. The transcription levels of the key genes involved in artemisinin biosynthesis were investigated using real time RT-PCR, revealing that cytochrome P450 monooxygenase (*CYP*71*AV*1) and cytochrome P450 oxidoreductase (*CPR*) expression were up-regulated in *A. annua* upon inoculation with strain YIM 63111 under certain conditions. The up-regulation of these genes was associated with the increased accumulation of artemisinin. These results suggest that endophytic actinobacteria effectively stimulate certain plant defense responses. Our data also demonstrate the use of *Pseudonocardia* sp. strain YIM 63111 as a promising means to enhance artemisinin production in plants.

## Introduction

The herb *Artemisia annua* L. is a member of the Asteraceae family that has been used in traditional Chinese medicine for the treatment of fever and malaria since ancient times. In 1971, the chemical compounds responsible for the antimalarial properties of the traditional medicine “qinghaosu” (artemisinin) were discovered by Chinese scientists [1]. Since then, artemisinin-based combination therapies (ACTs) recommended by the World Health Organization have been shown to be highly effective in preventing the infection and transmission of multi-drug resistant (MDR) malaria. However, the cost of artemisinin prevents its widespread use in malaria patients, as artemisinin is produced in trace amounts by the plant *A. annua* [2]. Synthetic antimalarial drugs and malaria vaccines are currently being developed but are years away from clinical use [3].

Due to the great importance of artemisinin, a significant effort has been made to enhance the production of artemisinin. Previous attempts to stimulate artemisinin synthesis involved the culture of *A. annua* plants in the presence of abiotic stresses such as cold, heat and UV irradiation [4], plant hormones [5] and exogenous methyl jasmonate [6]. In addition, oligogalacturonides [7], cerebroside in combination with nitric oxide [8], miconazole [9] and methyl jasmonate alone or in combination with β-cyclodextrins [9], [10] were used to treat hairy roots and cell cultures of *A. annua*. However, due to the cost and the limitation of low yields of artemisinin in *A. annua* tissue and cell cultures, the availability of artemisinin will continue to be dependent on extraction from whole plant cultures of *A. annua* [11].

Plants constitute a vast and diverse niche for endophytic organisms resulting in the development of closer biological associations between the plant and the endophytes than epiphytes or other soil organisms [12]. Evidence has also accumulated suggesting that secondary metabolites in plants actively take part in plant-microbe interactions. Endophytes colonize the internal tissue of the plant and are capable of triggering physiological plant responses [13], [14] and influencing the production of secondary metabolites in the host plant [15], [16]. Wang et al. purified a chemical elicitor from the extract of the endophytic fungus *Colletotrichum* sp., which could be used to stimulate the accumulation of artemisinin in *A. annua* hairy roots [17]. Artemisinin biosynthesis has also been shown to be enhanced in *A. annua* after treatment with *Piriformospora indica* or arbuscular mycorrhizal fungi [11], [18], [19]. A large number of endophytic actinomycetes have been isolated from *A. annua* [20]. However, *A. annua*-endophytic actinomycete interactions have only been rarely documented.

In the present study, the effect of endophytic actinomycete strains on the growth and accumulation of artemisinin in *A. annua* was carefully determined. Additionally, we analyzed the transcription levels of the genes amorpha-4,11-diene synthase (*ADS*) [21], cytochrome P450 monooxygenase (*CYP71AV1*) and cytochrome P450 oxidoreductase (*CPR*) [3], which are key regulatory genes in the biosynthesis of artemisinin, as well as the gene squalene synthase (*ASQS*) [22], [23], which is the key gene in the competing pathway diverting farnesyl diphosphate (FDP) to steroid synthesis.

## Results

### Effects of Endophytic Actinomycetes on the Growth of *A. annua* and Artemisinin Accumulation

In a preliminary assay, 6-day-old geminated *A. annua* seedlings were inoculated with the endophytic strains YIM 63654, YIM 63673, YIM 63342 YIM 63538 and YIM 63111. The plants were incubated for 66 days, and all treated and untreated seedlings were harvested to determine the plant height and artemisinin content. The height of the seedlings that were inoculated with strains YIM 63654, YIM 63673, YIM 63342 and YIM 63538 were similar to the non-inoculated control plants, whereas the growth of *A. annua* seedlings inoculated with strain YIM 63111 was significantly inhibited when compared with the control plants (Fig. S1). However, artemisinin production was significantly elevated upon inoculation with strain YIM 63111 (*P*<0.05). The artemisinin content was 2.57±0.18 mg g^−1^ dry weight (DW) compared to 1.99±0.09 mg g^−1^ DW that was observed in the control samples (Fig. S2). based on these results, strain YIM 63111 was selected for further experiments.

### Characterization of Strain YIM 63111

Pink aerial mycelium and yellow brown substrate mycelium were produced on yeast extract-malt extract (International *Streptomyces* Project, ISP 2) agar plates. Morphological observation of a 14-day-old culture of strain YIM 63111 revealed that both aerial and vegetative hyphae were abundant, well developed and fragmented. The mycelia displayed long spore chains containing more than 10 rod-shaped and smooth-surface spores (0.6–0.9×1.5–3.0 µm) (Fig. S3). These results suggested that strain YIM 63111 was morphologically similar to species within the genus *Pseudonocardia*. The nearly complete 16S rRNA gene sequence of strain YIM 63111 (1511 bp) was determined and was compared with the corresponding sequences of other bacterial strains in the GenBank database. Phylogenetic analysis based on 16S rRNA gene sequences revealed that strain YIM 63111 is a species of the genus *Pseudonocardia*. Neighbor-joining phylogenetic analysis showed that strain YIM 63111 exists within the same subclade as *Pseudonocardia alni* DSM 44104^T^ (99.8% similarity), *Pseudonocardia antarctica* DSM 44749^T^ (99.7% similarity) and *Pseudonocardia carboxydivorans* JCM 14827^T^ (99.9% similarity) (Fig. S4). Nonetheless, we observed various differences in the physiological characteristics of the *Pseudonocardia* species such as the carbon source and optimal growth temperature (Table S1).

### Strain YIM 63111 Colonization in Root

**Figure 1:**
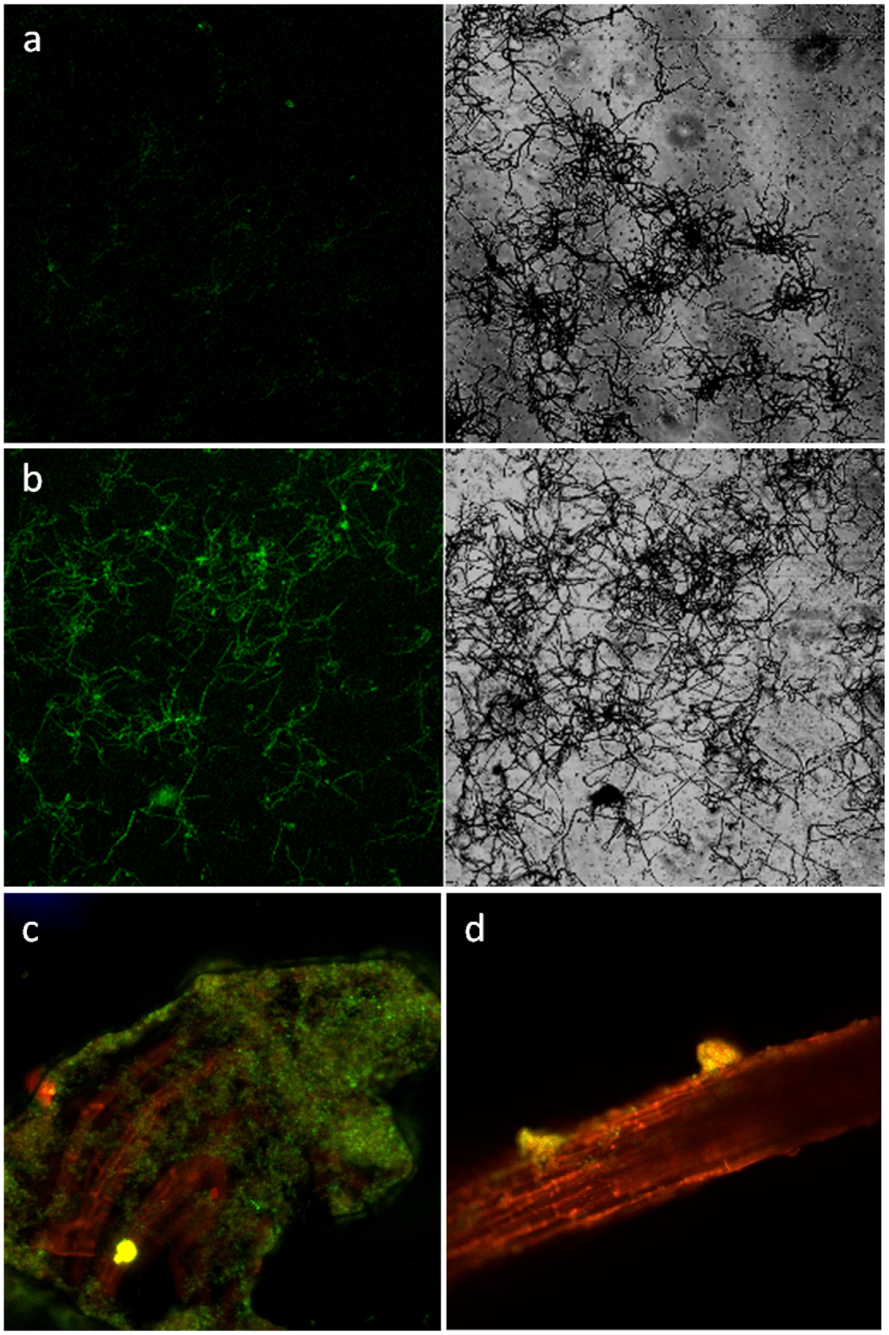
Laser-scanning confocal microscopy of strain YIM 63111 tagged with *egfp* for colonization analysis. a) Wild-type strain YIM 63111; b) *egfp*-tagged strain YIM 63111; c) Outer colonization of the root 14 days after inoculation; d) Outer and inner colonization of the root 14 days after inoculation. EGFP was excited with a 488-nm laser, and fluorescence was detected at 505–530 nm.

Positive transformants, which showed apramycin resistance, were generated by electroporation. The vector pIJ8660 used in this study does not contain a promoter region; however, the enhanced *green fluorescent protein* gene (*egfp*)-positive YIM 63111 transformants showed strong *green fluorescent protein* (GFP) fluorescence when visualized by fluorescence microscopy (Leica DMI6000B) and confocal laser-scanning microscopy (Zeiss LSM510 META) when compared with the control wild-type strain YIM 63111 (Fig. 1a & 1b). Microscopy results showed that YIM 63111 colonized the inner tissues of seedling roots and lived intercellularly (Fig. 1c & 1d). Microscopic analysis also revealed that colonization occurred via the emerging lateral roots (Fig. 1d). These results provide visual evidence for the colonization of *A. annua* by strain YIM 63111, which complement the results (Fig. S5) from the re-isolation experiments after inoculation.

### Effect of YIM 63111 Inoculation on *A. annua* Under Sterile Conditions

We observed that the growth of 64-day-old *A. annua* seedlings inoculated with YIM 63111 at concentrations of 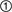
3.1×10^8^, 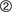
6.25×10^7^, 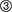
1.25×10^7^, 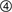
2.5×10^6^, 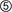
5.0×10^5^, 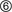
1.0×10^5^ and 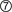
2.0×10^4^ CFU ml^−1^ were inhibited showing a stunted root system. The root length of the infected plants was 0.8–6.3 cm compared with roots that averaged 9.5 cm in length in the non-inoculated plants (*P*<0.01, Fig. S6 and S7). However, the inhibitory effect of YIM 63111 infection was not significant when the concentration of the inoculum was reduced to 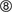
4.0×10^3^, 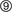
750∼800 and 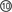
150∼200 CFU ml^−1^, as the root length of these samples was approximately 8.5 cm.

In the re-isolation experiments, a bacterial strain that was morphologically identical to the YIM 63111 strain was retrieved from the surface-sterilized seedlings after inoculation with strain YIM 63111 at concentrations ranging from 3.1×10^8^ to 4.0×10^3^ CFU ml^−1^ (Fig. S5).

**Figure 2:**
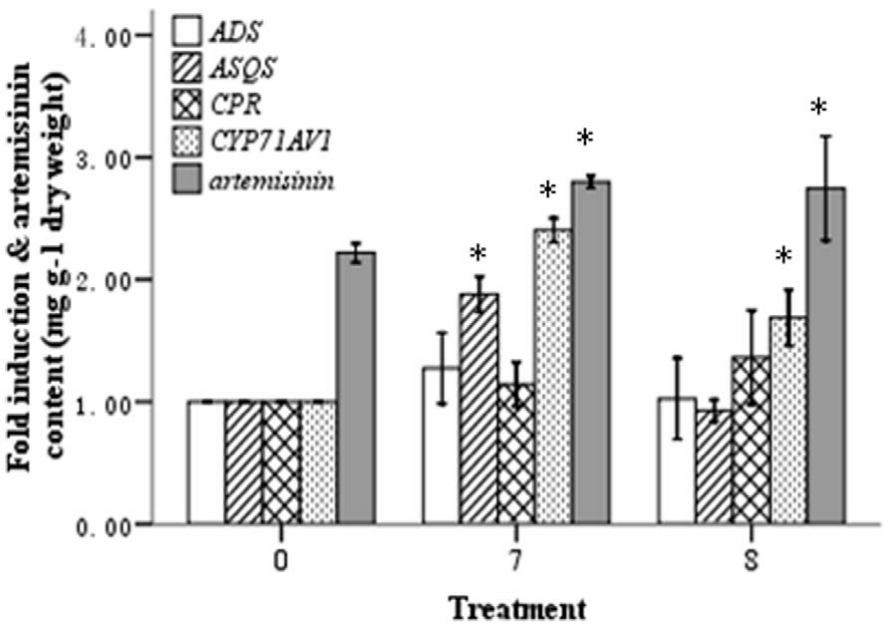
Transcript abundance of the *ADS*, *ASQS*, *CPR* and *CYP71AV1* genes and artemisinin content in *A*. *annua* plants grown for 64 days after YIM 63111 inoculation compared with untreated plants (No. 0). Nos. 7 & 8 indicate the seedlings that were inoculated with serially diluted bacterial suspensions, 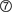 2.0×10^4^ CFU ml^−1^, 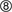 4.0×10^3^ CFU ml^−1^. * indicates a significant difference, and the error bars represent the standard deviation.

According to the growth characteristics and the results of the re-isolation experiments, untreated *A. annua* samples and samples treated with actinobacteria suspensions at a concentration of 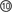
2.0×10^4^ and 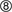
4.0×10^3^ CFU ml^−1^ were used to determine the transcript levels of key genes in the artemisinin biosynthesis pathway and to measure the content of artemisinin. The results from colony counting experiments showed that the density of YIM 63111 upon harvest of the plants was (1.26±0.38)×10^3^ CFU g^−1^ when plants were inoculated with 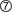
2.0×10^4^ and 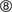
4.0×10^3^ CFU ml^−1^. Gene expression analysis revealed that the inoculation of *A. annua* seedlings with strain YIM 63111 at a concentration of 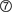
2.0×10^4^ CFU ml^−1^ caused a significant increase in *CYP71AV1* and *ASQS* transcript levels by 2.4- and 1.9-fold, respectively, when compared with the non-inoculated control (*P*<0.01). In the seedlings treated with 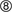
4.0×10^3^ CFU ml^−1^ of strain YIM 63111, *CYP71AV1* transcript levels were 1.7-fold higher than the non-inoculated control plants (*P*<0.01). These results are in contrast to the transcript levels of *ASQS*, which remained unchanged upon strain YIM 63111 inoculation. In both treatments 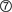 and 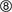 the transcript levels of *ADS* and *CPR* did not show significant variation. Furthermore, quantitative analysis of artemisinin content showed that artemisinin production was significantly enhanced by 35.1% and 32.6% upon inoculation with strain YIM 63111 at concentrations of 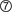
2.0×10^4^ and 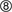
4.0×10^3^ CFU ml^−1^, respectively (*P*<0.05) (Fig. 2).

### Effect of the Inoculation of Strain YIM 63111 on *A. annua* Under Greenhouse Conditions

*A. annua* plants grown for 74 days post-inoculation with strain YIM 63111 and non-inoculated controls were harvested, and the heights and fresh weights of their shoots were measured. The results showed that YIM 63111 inoculation did not influence the growth of the plants (Fig. S8).

**Figure 3:**
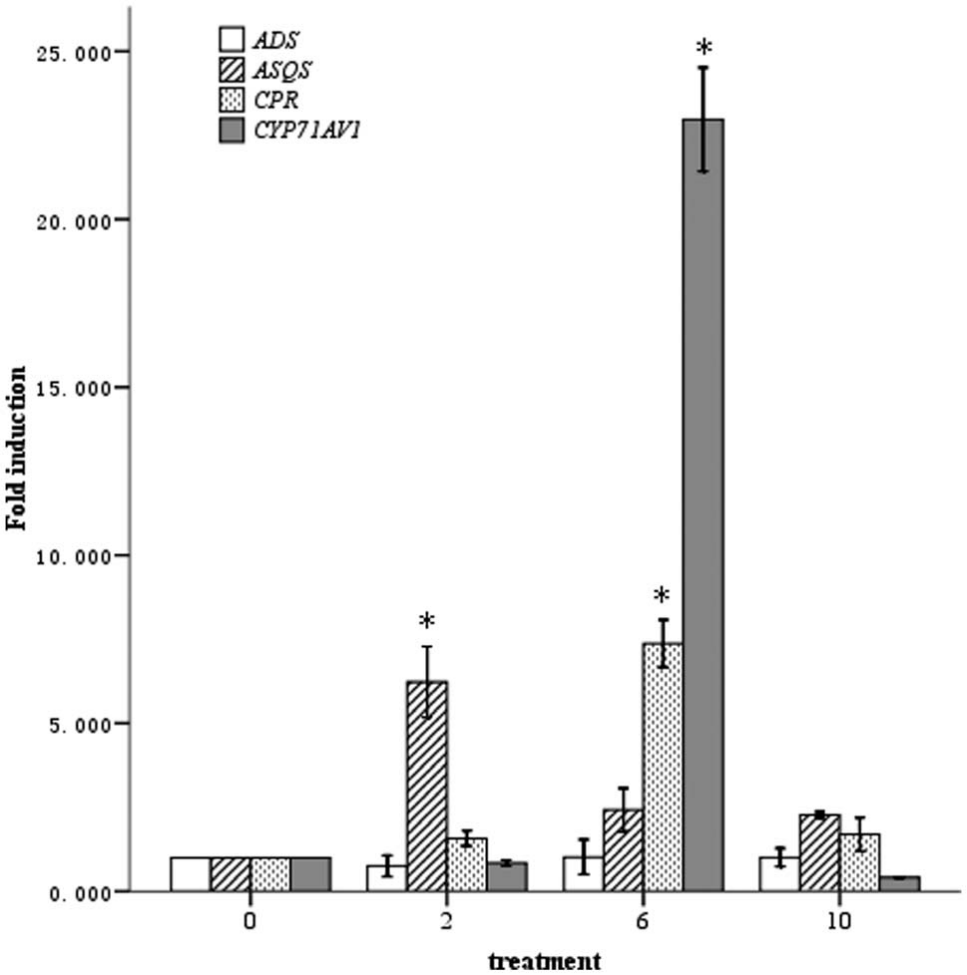
Transcript abundance of the *ADS*, *ASQS*, *CPR* and *CYP*71*AV*1 genes in *A*. *annua* plants grown for 74 days after YIM 63111 inoculation compared with untreated plants (No. 0). Nos. 2, 6 and 10 indicate the seedlings that were inoculated with serially diluted bacterial suspensions, 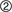 3.68×10^8^, 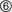 5.90×10^5^ and 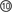 900∼1.0×10^3^ CFU ml^−1^. * indicates a significant difference (*P*<0.01), and the error bars represent the standard deviation.

**Figure 4:**
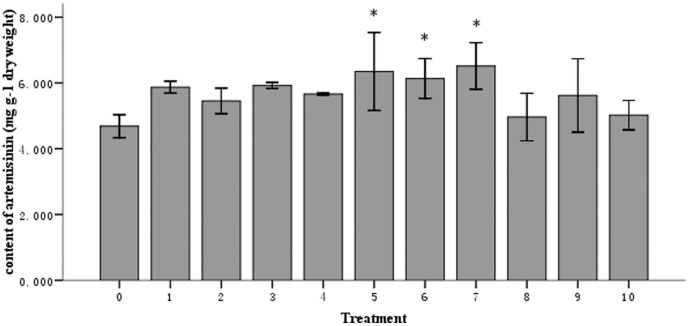
Artemisinin content in *A*. *annua* plants grown for 74 days after YIM 63111 inoculation compared with untreated plants (No. 0). Nos. 1–10 indicate the seedlings that were inoculated with serially diluted bacterial suspensions: 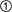 1.84×10^9^, 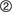 3.68×10^8^, 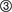 7.38×10^7^, 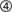 1.48×10^7^, 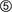 2.96×10^6^, 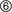 5.90×10^5^, 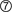 1.18×10^5^, 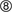 2.36×10^4^, 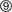 4.72×10^3^ and 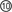 900∼1.0×10^3^ CFU ml^−1^. * indicates a significant difference (*P*<0.05, and the error bars represent the standard deviation.

Samples that were non-inoculated or that were inoculated with strain YIM 63111 at concentrations of 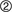
3.68×10^8^, 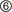
5.90×10^5^ and 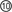
900−1.0×10^3^ CFU ml^−1^ were used for gene expression analysis. When a higher concentration inoculum (treatment 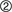) was applied, *ASQS* transcript levels were induced 6.2-fold, whereas the transcript levels of *ADS*, *CPR* and *CYP71AV1* remained unchanged. Furthermore, artemisinin content did not show significant variation relative to the control samples (Figs. 3 & 4). When the plants were treated with a bacterial suspension at a medium concentration (treatment 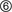), the artemisinin biosynthesis pathway was more strongly induced, as a 7.4-fold increase was observed for *CPR* transcript levels, and a 23-fold increase was observed for *CYP71AV1* transcript levels (*P*<0.01). However, this treatment group did not show significant changes in the *ADS* and *ASQS* transcript levels (Fig. 3). Consistent with these results, artemisinin content was increased by 30.9% in this treatment group relative to the non-inoculated control group, as this YIM 63111-inoculated group had an average of 6.14 mg g^−1^ of dry weight artemisinin (Fig. 4). In addition to treatment group 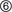, plants inoculated with strain YIM 63111 at concentrations of 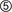
2.96×10^6^ and 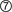
1.18×10^5^ CFU ml^−1^ also showed increases in artemisinin biosynthesis by 35.5% and 39.0%, respectively. These increases accounted for 6.35 and 6.52 mg g^−1^ of the dry weight of these samples, respectively (Fig. 4). Inoculation of YIM 63111 at a concentration of 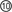
900∼1.0×10^3^ CFU ml^−1^ did not result in significant gene induction relative to the control plants, and no changes were observed in artemisinin content. Additionally, no changes were observed in gene expression or artemisinin content in treatment groups 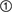, 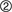, 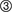, 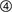, 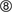 and 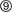
(Figs. 3 & 4).

## Discussion

To our knowledge, this study represents the first report successfully using EGFP expression in non-*Streptomyces* endophytic actinomycete colonization research. The observations in this study confirmed that strain YIM 63111 could actively colonize *A. annua*. Although plasmid pIJ8660 does not contain any promoter region, the *egfp* gene showed microscopically observable expression levels in the positive transformants. We suspect that the pIJ8660 plasmid inserted within the bacterial chromosome at a position that facilitated its expression. However, further research is required to determine the validity of our suspicions and will be useful for studies involving the genetic manipulation of *Pseudonocardia*-strains.

In this study, we observed that strain YIM 63111 stunted the root development and subsequently inhibited the growth of *A. annua* seedlings grown under sterile conditions when inoculated at higher concentrations (>1.0×10^5^ CFU ml^−1^). However, strain YIM 63111 did not show effects on the growth of *A. annua* under greenhouse conditions. *Paenibacillus polymyxa*, which has been characterized as a plant growth-promoting bacterium, has been reported to cause root stunting and reduced plant growth in the absence of biotic or abiotic stress [24], [25]. Similarly, we think that *Pseudonocardia* sp. strain YIM 63111 caused biotic stress to the *A. annua* seedlings under sterile conditions in the absence of competition from other soil microorganisms. To our knowledge, neither pathogenic nor harmful effects on plants were observed for members of the genus *Pseudonocardia*, although the closest phylogenetic neighbor *P. alni* has also been shown to associate with higher order plants [26]. This phenomenon indicates that the interaction between endophytic bacteria and plants varies in a seamless manner from a mutualistic to a parasitic relationship, which is similar to previous reports for some endophytic fungi [27]. These results support the idea that endophytes possess many pathogenic virulence factors, and the asymptomatic colonization by an endophyte is a delicate balance of antagonism between the host and the endophyte(s) [28].

Artemisinin is a phytoalexin, which has allelopathic effect and defends plants against herbivores and exogenous microbes [29], [30]. Both arbuscular mycorrhizal fungi and endophytic fungi have been reported to enhance artemisinin accumulation [18]. *A. annua* showed healthy growth and increased secondary metabolite content post-treatment with the endophytic fungus *P. indica* [19], [31], [32]. Wang et al. [17] reported an increase in artemisinin content in hairy roots of *A. annua* from 0.8 mg g^−1^ DW to 1 mg g^−1^ DW following an elicitor treatment with mycelial extracts from the endophytic fungus *Colletotrichum* sp. *Pseudonocardia* species have a wide host range [33]–[35] and are abundant in *A. annua* associations [20]. The results obtained in this study represent the first description of the ability of a member of the genus *Pseudonocardia* to increase artemisinin production.

The present study shows that strain inoculation up-regulates the expression of the genes *CYP71AV1* and *CPR*, which consequently increases the biosynthesis of artemisinin. CYP71AV1 and its redox partner CPR are key contributors to the conversion of amorpha-4,11-diene to artemisinic acid [3]. Our results indicate that the stimulatory effect of strain YIM 63111 on artemisinin production could be due to the up-regulation of the *CYP71AV1* and *CPR* genes. The transcript level of the *CYP71AV1* gene and the artemisinin content have been reported to be notably higher in *A. annua* cell cultures treated with methyl jasmonate relative to untreated cultures [9], [10]. Methyl jasmonate is considered a key compound in the signal transduction pathway and methyl jasmonate plays a role in several plant responses [36]. Moreover, the endophytic actinomycete strains that belong to the genus *Streptomyces* and *Nocardioides* have been reported to activate systemic acquired resistance (SAR) or the jasmonate/ethylene (JA/ET) pathway [37]. Whether the *Pseudonocardia* sp. strain YIM 63111 triggers plant responses through methyl jasmonate signaling will require further experimentation. Additionally, previous reports have indicated that abiotic stress such as low temperature incubation leads to elevated artemisinin content within 48 h, which was further supported by observed increases in the transcript levels of the *ADS* and *CYP71AV1* genes and the stable expression of the *CPR* gene [4]. These results suggest that the *CYP71AV1* gene responds to both biotic and abiotic factors. The calcium-calmodulin signal transduction pathway has been suggested to play a role in the up-regulation of the transcript levels of the *ADS* and *CYP71AV1* genes under low temperature stress [38]. Whether the induction effect of abiotic and biotic elicitors on *ADS*, *CYP71AV1* and *CPR* gene expression act through similar mechanisms will require further research.

In *A. annua,* FDP serves as a common precursor for the synthesis of artemisinin and steroids. Thus, the enzymes ADS and ASQS directly compete for FDP to determine whether FDP gets funneled through the artemisinin or the steroid synthesis pathway [23]. The inoculation of *A. annua* seedlings with strain YIM 63111 at higher concentrations (treatment 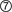 under sterile conditions and treatment 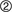 under greenhouse conditions) resulted in an increase in the transcript level of the *ASQS* gene. However, the transcript level of the *ADS* gene and the artemisinin content in these samples did not change. Yang et al. [39] observed decreased levels of ASQS mRNA and artemisinin content in transgenic plants that express a genomic-integrated antisense squalene synthase gene. These observations reflect that redirecting the carbon flux by regulating gene expression often leads to complex results. Results of this study show that *CYP71AV1* gene expression in seedlings grown under greenhouse conditions increased to a greater extent than plants cultured under sterile conditions. In addition, as previous reports [4], our results also show that the transcript level of the *CPR* gene remained unchanged in seedlings grown under sterile conditions, whereas *A. annua* inoculated with strain YIM 63111 grown under greenhouse conditions showed an up-regulation of *CPR* gene expression. Collectively, these results suggest that the inducible transcription of the *CPR* and *CYP71AV1* genes are most likely connected with seedling development.

Taken together, results obtained from our study suggest that during plant-microbe interactions, the recognition of the endophyte YIM 63111 by *A. annua* triggers a series of plant defense responses resulting in a shift in plant metabolism. However, the mechanism responsible for the observed reduction in growth and increase in production of artemisinin in *A. annua* upon inoculation with strain YIM 63111 warrant further research.

To the best of our knowledge, this is the first study reporting the induction of artemisinin in *A. annua* by an endophytic actinomycete. The results obtained in this study reveal a role for the regulation of artemisinin biosynthesis in response to actinomycete colonization and establish a relationship between endophytic actinomycetes and *A. annua* host plants.

## Materials and Methods

### Ethics Statement

Because *A. annua* is not a protected species, no specific permits were required for sampling.

### Endophytic Actinomycete Cultures

The endophytic actinomycete strains YIM 63654 (*Micromonospora* sp.), YIM 63673 (*Streptosporangium* sp.), YIM 63342 (*Streptomyces* sp.), YIM 63538 (*Micromonospora* sp.) and YIM 63111 (*Pseudonocardia* sp.) were maintained on ISP 2 agar [40]. All cultures used in this study were isolated previously from surface-sterilized *A. annua* samples, as previously described [20].

### Morphological Characteristics and Phylogenetic Analysis of Strain YIM 63111

The morphological characteristics of strain YIM 63111 including spore-chain morphology, spore size and surface ornamentation were assessed by light (BH-2; Olympus) and scanning electron microscopy (Philips XL30; ESEM-TMP) of 14-day-old cultures grown on ISP 2 medium [40]. Extraction of genomic DNA, PCR amplification and sequencing of the 16S rRNA gene were performed as described by Li et al. [41]. The resulting 16S rRNA gene sequences were compared with available 16S rRNA gene sequences from GenBank using the BLAST program (http://www.ncbi.nlm.nih.gov/BLAST/) to determine an approximate phylogenetic affiliation. Phylogenetic trees were constructed by the neighbor-joining tree-making algorithms by using the software package MEGA version 4.0 [42]. Physiological tests such as growth at different temperatures and different salinities were performed by culturing the strains in tryptic soy broth (TSB) basal medium. Other physiological characteristics were assessed as previously described [43], [44].

### Inoculation of *A. annua* Seedlings Grown Under Sterile Conditions with Actinobacterial Strains

Strains YIM 63654, YIM 63673, YIM 63342 YIM 63538 and YIM 63111 were grown on ISP 2 agar by incubating the samples at 28°C for 8 days. Three milliliters of sterile distilled water was then added to the plates before they were scraped gently with a loop to loosen the spores and the mycelia. This suspension was then transferred into a centrifuge tube and was centrifuged at 7,000 *g* for 5–10 min. The supernatant was discarded, and the pellet was suspended in sterile distilled water. The bacterial cell suspension was diluted to a final concentration of 5.0×10^4^ CFU ml^−1^. For the inoculation experiments involving strain YIM 63111, the bacterial suspension was serially diluted to 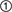 3.1×10^8^, 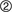
6.25×10^7^, 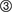
1.25×10^7^, 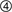
2.5×10^6^, 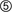
5.0×10^5^, 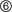
1.0×10^5^, 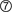
2.0×10^4^, 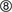
4.0×10^3^, 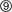
750∼800 and 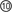
150∼200 CFU ml^−1^.

*A. annua* (F38#) seeds were surface-sterilized by immersing the seeds in 70% ethanol for 1 min followed by five washes with sterile distilled water. Then, the seeds were rinsed in 5% (available Cl^-^) NaOCl for 10 min and finally washed five times with sterile distilled water. Seeds were then placed on phytohormone-free Murashige and Skoog (MS) medium supplemented with 3% sucrose and solidified with 0.7% agar. Then, seeds were placed at 4°C overnight in the dark and then were transferred to a growth chamber at 25°C exposed to a 16-h light (*ca*. 2,000 lux)/8-h dark cycle. Some of the sterilized seeds were crushed and placed onto ISP 2 agar medium with incubation at 28°C for 1 week, and no colonies were observed on these plates.

The bacterial suspension (20 µl) was dropped onto the surface of the agar medium around the base of each shoot of 6-day-old germinated *A. annua* seedlings. In untreated controls, 20 µl of sterile distilled water was used. Three seedlings were grown in each glass bottle. Each treatment contained nine glass bottles of cultures. Whole seedlings without the roots were used for the quantification of artemisinin content.

### Re-isolation of Inoculated Strains from *A. annua* Seedlings Grown Under Sterile Conditions

Seedlings were randomly harvested from three bottles in each treatment group (one seedling from each bottle). Samples were surface-sterilized as follows: 5% (available Cl^-^) NaOCl for 2 min, three washes with sterile H_2_O, 70% (v/v) ethanol for 2 min, and then three washes with sterile H_2_O. Surface sterilization was verified as described in the procedures for strain isolation. Seedlings were cut into segments, spread onto ISP 2 agar without antibiotics, and incubated at 28°C for several days.

### Inoculation of *A. annua* Grown Under Greenhouse Conditions with Strain YIM 63111

Surface-sterilized *A. annua* (F38#) seeds were sown into 32 wells each with a diameter of 6 cm and a height of 5.5 cm each well and were incubated in a greenhouse located at Southwest Forestry University. The temperature in the greenhouse ranged from 20 to 28°C, and the relative humidity was 70%. The substratum was a mixture of peat soil and perlite, which was sterilized at 121°C for 2 hours. Nine-day-old seedlings were grown to an identical phase and then separated into separate pots. Bacterial suspensions at concentrations of 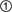
1.84×10^9^, 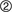
3.68×10^8^, 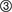
7.38×10^7^, 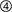
1.48×10^7^, 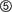
2.96×10^6^, 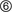
5.90×10^5^, 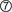
1.18×10^5^, 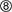
2.36×10^4^, 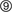
4.72×10^3^ and 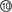
900∼1.0×10^3^ CFU ml^−1^ were prepared as described above, and 0.5 ml of the prepared inoculum was poured onto the surface of the substratum around the seedlings. Sterile water was used as a control inoculum. Each treatment included 16 plants divided into 16 (4×4) pots in triplicate. The plugs were distributed in a randomized manner, and their positions were changed once a week.

Three leaves under the third visible leaf from the apex were harvested, pooled, and used for RNA extraction. After fresh weight determination, all leaves of the 16 samples were mixed and were used for the quantification of artemisinin content.

### Extraction and quantification of artemisinin

Leaves of seedlings were dried to a constant weight in an oven at 50°C and were ground into a fine powder. Forty milligrams of dry powder (1 g used in the greenhouse experiment) was placed into a flask that contained 30 ml petroleum ether (30–60°C). The powder was extracted overnight, after which the extract was collected and an additional 30 ml of petroleum ether was added to the bottle. The filtrates were collected and were evaporated with a rotary evaporator. The residue was dissolved in methanol and was diluted to a total volume of 1 ml (5 ml for the greenhouse experiment samples). For the quantification of artemisinin, 10 µl of the sample solution was injected into an HPLC instrument (Agilent 1100 series) with a Phenomenex Luna C18 (2) column (250 mm×4.6 mm, 5 µm particle size). An acetonitrile-water mixture (50∶50, v/v) was used for the mobile phase at a 1 ml min^−1^ flow rate, 30°C column temperature, and 210-nm wavelength for detection. Artemisinin content [mg g^−1^ DW] was calculated from the artemisinin amount (µg)/sampling volume (10 µl)×total volume (1000 µl or 5000 µl)/sample weight (40 mg or 1 g). To prepare the standard control samples, 4 mg and 8 mg of artemisinin was dissolved in 10 ml of methanol. A standard curve was plotted by using aliquots of 2, 4, 8, 12, 16 and 20 µl of 0.4 mg ml^−1^ artemisinin solution and 10, 15, 20 µl of 0.8 mg ml^−1^ artemisinin solution was individually injected into the sampling outlet of the HPLC instrument for quantification by the procedure described above.

### Relative Quantification of Gene Expression

Total RNA was extracted from 30 mg of fresh leaves of *A. annua* using the SV total RNA isolation system (Promega). Reverse transcription reactions of the total RNA and real-time fluorescent PCR were performed using the SYBR PrimeScript & RT-PCR Kit (TaKaRa). The 18S rRNA gene of *A. annua* was used as an internal control to normalize experimental gene expression. The primers used were designed according to the cDNA sequences of *A. annua* for gene expression analysis and are as follows: 18S rRNA gene (forward primer, F) 5′ GCA ACA AAC CCC GAC TTC TG 3′ and (reverse primer, R) 5′ TGC GAT CCG TCG AGT TAT CA 3′; *ADS* (F) 5′ GTC GAA TGG GCT GTC TCT GC 3′ and (R) 5′ TTC TTT CTT GCT CGG CCT TG 3′; *CPR* (F) 5′ GAT AAC GAA GAC GGG ACA CCA 3′ and (R) 5′ GCT CAA AAC ATC GGC ATA GGA 3′; *CYP71AV1* (F) 5′ TGG TTC TGC CAA GAG AGT GC 3′ and (R) 5′ GGT CCC TAT TTA TCG CAA AGA CG 3′; *ASQS* (F) 5′ CGG ACT AAA ACA ATG GCT GAT G 3′ and (R) 5′ GTG ATG GTC GTT TGG GCA TT 3′ [39]. The reaction was conducted using the real-time PCR system ABI PRISM 7000. Data were acquired on the SYBR-green channel for both quantitative and melting analysis. Cycle threshold (Ct) values were obtained from raw fluorescence data using quantitative analysis and the ABI Prism 7000 SDS software. Serial 5-fold (for 18S rRNA gene) and 2-fold (for other genes) dilutions of the cDNA products were made, and the dilutions were used as the template for each real-time reaction. Reactions were performed in 96-well plates, and standard curves were generated based on the least-squares linear regression method by plotting the Ct values against the relative template amount. Based on the standard curve, the relative template amounts for each sample were obtained, and then the relative gene expression levels were calculated by comparing the samples with the controls. The results are presented as the mean of three independent experiments. For each experiment, we assigned triplicate QPCR reactions for each sample where the RNA was pooled from leaves of five randomly selected seedlings.

### Genetic Transformation of Strain YIM 63111 with GFP

Strain YIM 63111 was transformed with *egfp* using the vector pIJ8660 [7.7 kb,*int*/*att*P(ΦC31),*aac(3)IV*,*egfp*,*ori*(pUC18),*ori*T_RK2_] [45]. Competent *Escherichia coli* ET12567 cells containing the helper plasmid pUZ8002 were transformed with pIJ8660 DNA. Positive transformants were used for intergenetic recombination with strain YIM 63111 according to the protocol described in *Practical Streptomyces Genetics* [46].

Fluorescent transformants were selected on mannitol-soy flour agar plates supplemented with 50 µg ml^−1^ apramycin, and *EGFP* expression was assessed by fluorescence microscopy (Leica DMI6000B) using the appropriate filters (450–490 nm) and confocal laser-scanning microscopy (Zeiss LSM510 META).

### *A. annua* Inoculation and Laser-scanning Confocal Microscopy

*A. annua* seedlings were either inoculated with *egfp*-tagged strain YIM 63111 or were not inoculated as described in the section entitled “Inoculation of *A. annua* seedlings grown under sterile conditions with actinobacterial strains”. The actinobacterial suspensions contained 8.0×10^7^ spores. Fourteen days after inoculation, root colonization was observed by confocal laser-scanning microscopy (Zeiss LSM510 META) using a 20× Plan-Neofluar (0.5 numerical aperture) objective lens. EGFP was excited with a 488-nm laser, and fluorescence was detected at 505–530 nm. Images were acquired using the following settings: 488-nm argon laser with a 505–530 bandpass emission filter and a 560 longpass emission filter.

### Statistical Analysis

One-way analysis of variance was conducted for each parameter studied. Tukey’s post-hoc multiple mean comparison test was used to determine the significance of the differences observed between the samples. A *p*-value less than 0.05 was considered statistically significant. All statistical analyses were performed using the Statistical Package for Social Sciences (version 15.0).

### Nucleotide Sequence Accession Number

The 16S rRNA gene sequence of strain YIM 63111 has been deposited in GenBank under the accession number FJ817376.

## Supporting Information

Figure S1 The height of *Artemisia annua* plants grown for 66 days after endophytic strains inoculation compared with untreated plants (No. 1). * indicates significant differences (*P*<0.05). Error bars represent standard deviation.
(TIF)

Figure S2 Artemisinin content in *A*. *annua* plants grown for 66 days after endophytic strains inoculation compared with untreated plants (No. 1). * indicates significant differences (*P*<0.05), error bars represent standard deviation.
(TIF)

Figure S3 Scanning electron micrograph of strain YIM 63111 grown on ISP 2 agar medium for 2 weeks at 28°C. Bar, 10 µm.
(TIF)

Figure S4 Phylogenetic relationships between strain YIM 63111 and other closely related *Pseudonocardia* species based on the 16S rRNA gene sequences. The branching pattern was generated by the neighbour-joining method. Bootstrap values (expressed as percentages of 1000 replications) of above 50% are shown at branch points. Bar, 0.005 substitutions per nucleotide position.
(TIF)

Figure S5Re-isolation of strain from inoculated *A*. *annua* seedlings. From left to right depict the re-isolation results obtained from seedlings which were inoculated with bacterial suspensions: 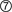
2.0×10^4^, 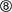
4.0×10^3^, 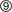
750∼800 and 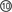
150∼200 CFU ml^−1^.(TIF)

Figure S6Morphological comparison of *A. annua* grown for 64 days after YIM 63111 inoculation with untreated plant. Nos. 1–10 indicate the seedlings which were inoculated with serially diluted strain suspensions: 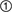
3.1×10^8^, 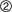
6.25×10^7^, 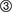
1.25×10^7^, 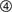
2.5×10^6^, 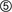
5.0×10^5^, 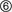
1.0×10^5^, 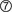
2.0×10^4^, 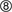
4.0×10^3^, 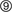
750∼800 and 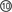
150∼200 CFU ml^−1^.(TIF)

Figure S7The root length of *A. annua* plants grown for 64 days after endophytic strains inoculation compared with untreated control (No. 0). * indicates significant differences (*P*<0.01), Error bars represent standard deviation. Nos. 1–10 indicate the seedlings that were inoculated with serially diluted bacterial suspensions: 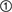
3.1×10^8^, 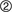
6.25×10^7^, 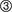
1.25×10^7^, 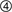
2.5×10^6^, 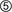
5.0×10^5^, 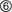
1.0×10^5^, 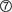
2.0×10^4^, 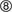
4.0×10^3^, 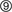
750∼800 and 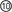
150∼200 CFU ml^−1^.(TIF)

Figure S8The heights and fresh weights of *A. annua* shoots grown for 74 days after YIM 63111 inoculation compared with untreated plants (No. 0). Nos. 1–10 indicate the seedlings that were inoculated with serially diluted bacterial suspensions: 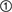
1.84×10^9^, 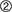
3.68×10^8^, 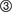
7.38×10^7^, 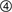
1.48×10^7^, 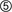
2.96×10^6^, 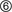
5.90×10^5^, 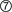
1.18×10^5^, 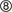
2.36×10^4^, 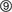
4.72×10^3^ and 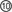
900∼1.0×10^3^ CFU ml^−1^. Error bars represent standard deviation.(TIF)

Table S1 Different characteristics of strain YIM 63111 and its closely related *Pseudonocardia* species.
(DOC)
