# Supplementary figures and images for: An Endophytic Pseudonocardia Species Induces the Production of Artemisinin in Artemisia annua
Source: PLoS One. 2012 Dec 12;7(12):e51410. doi: 10.1371/journal.pone.0051410 (PMC3520919; doi:10.1371/journal.pone.0051410)

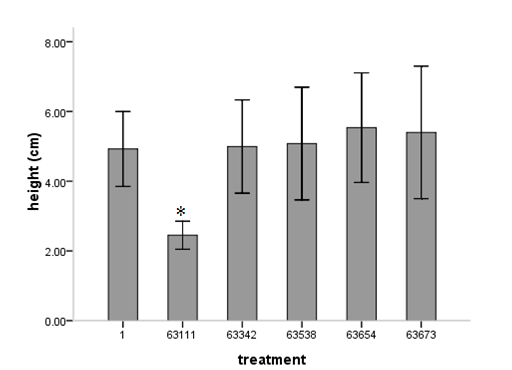

Supplement: Figure S1 — The height of Artemisia annua plants grown for 66 days after endophytic strains inoculation compared with untreated plants (No. 1). * indicates significant differences (P<0.05). Error bars represent standard deviation. (TIF) [file pone.0051410.s001.tif]

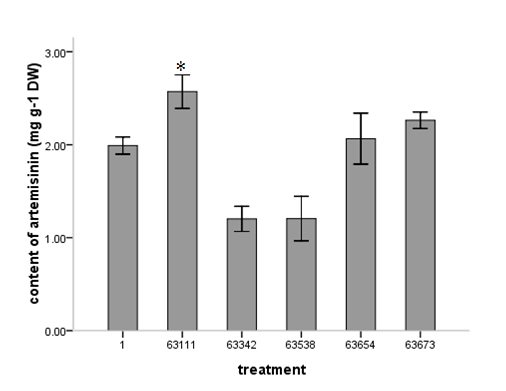

Supplement: Figure S2 — Artemisinin content in A. annua plants grown for 66 days after endophytic strains inoculation compared with untreated plants (No. 1). * indicates significant differences (P<0.05), error bars represent standard deviation. (TIF) [file pone.0051410.s002.tif]

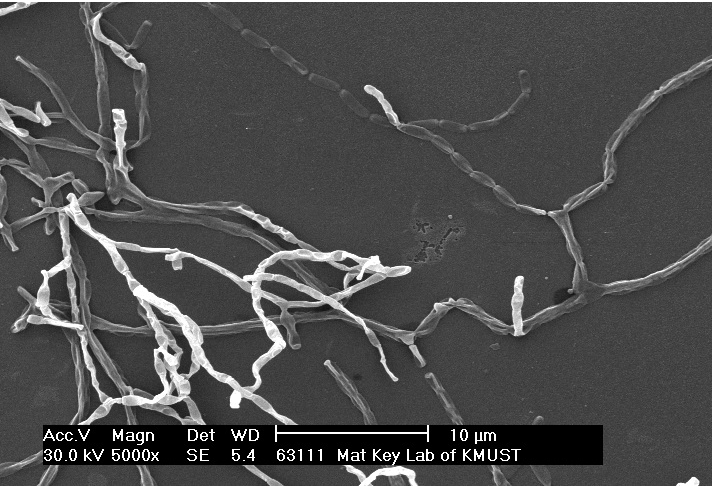

Supplement: Figure S3 — Scanning electron micrograph of strain YIM 63111 grown on ISP 2 agar medium for 2 weeks at 28°C. Bar, 10 µm. (TIF) [file pone.0051410.s003.tif]

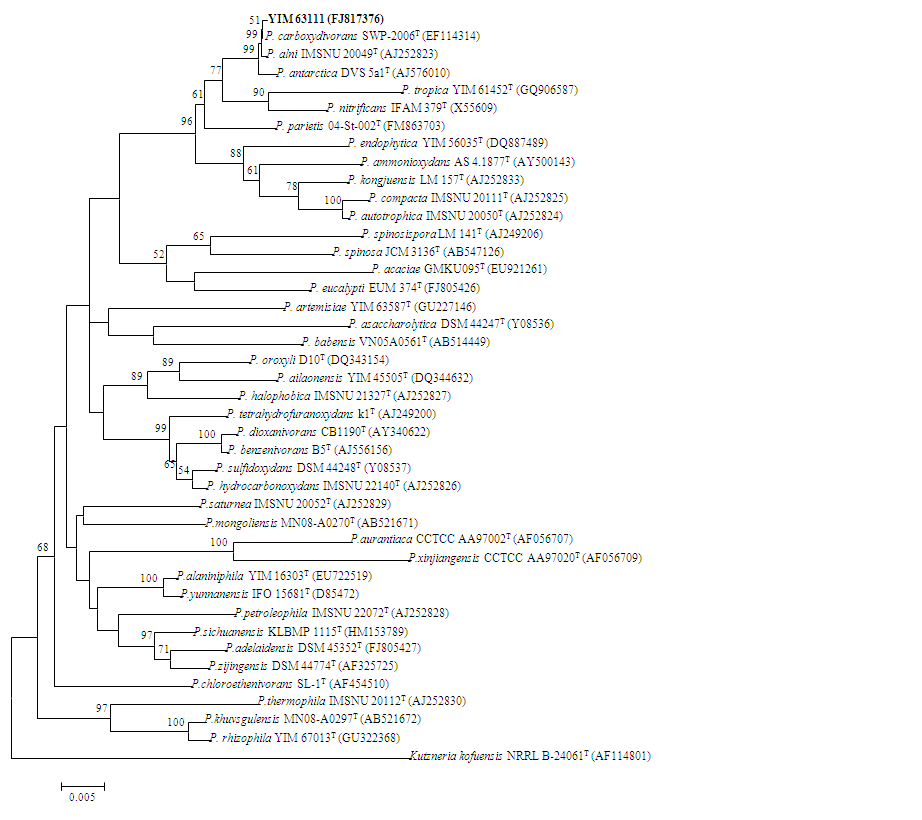

Supplement: Figure S4 — Phylogenetic relationships between strain YIM 63111 and other closely related Pseudonocardia species based on the 16S rRNA gene sequences. The branching pattern was generated by the neighbour-joining method. Bootstrap values (expressed as percentages of 1000 replications) of above 50% are shown at branch points. Bar, 0.005 substitutions per nucleotide position. (TIF) [file pone.0051410.s004.tif]

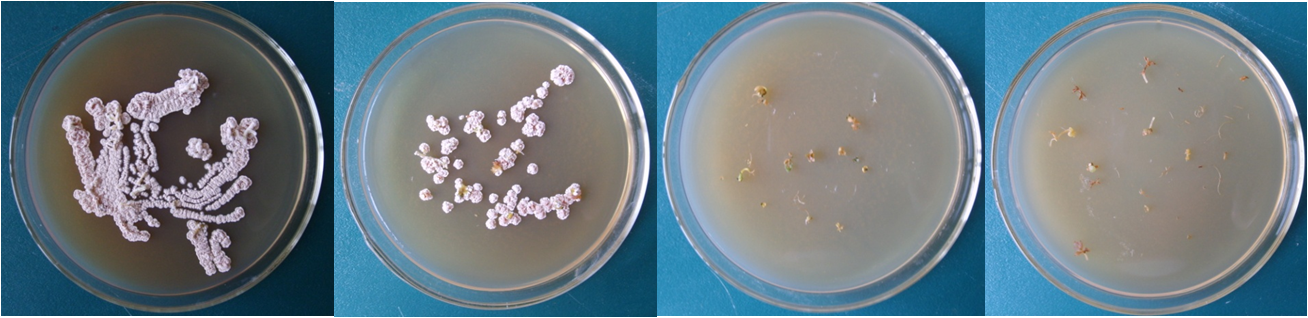

Supplement: Figure S5 — Re-isolation of strain from inoculated A. annua seedlings. From left to right depict the re-isolation results obtained from seedlings which were inoculated with bacterial suspensions: 2.0×10^4^, 4.0×10^3^, 750∼800 and 150∼200 CFU ml−1. (TIF) [file pone.0051410.s005.tif]

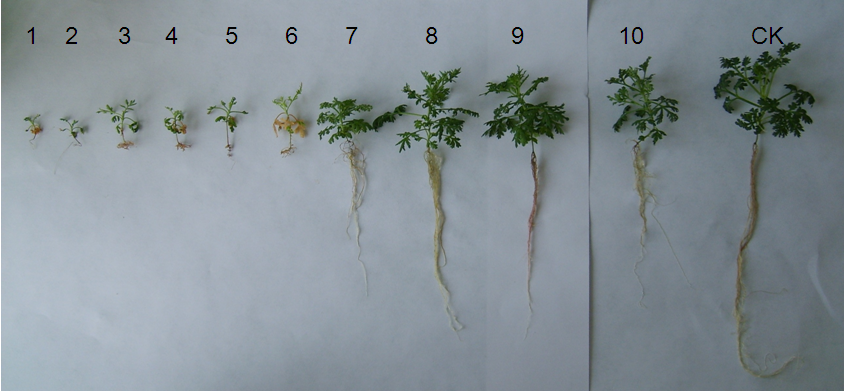

Supplement: Figure S6 — Morphological comparison of A. annua grown for 64 days after YIM 63111 inoculation with untreated plant. Nos. 1–10 indicate the seedlings which were inoculated with serially diluted strain suspensions: 3.1×10^8^, 6.25×10^7^, 1.25×10^7^, 2.5×10^6^, 5.0×10^5^, 1.0×10^5^, 2.0×10^4^, 4.0×10^3^, 750∼800 and 150∼200 CFU ml−1. (TIF) [file pone.0051410.s006.tif]

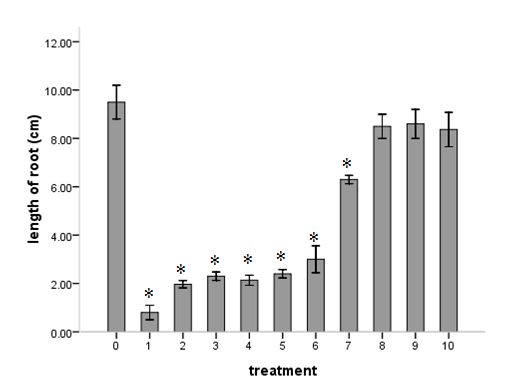

Supplement: Figure S7 — The root length of A. annua plants grown for 64 days after endophytic strains inoculation compared with untreated control (No. 0). * indicates significant differences (P<0.01), Error bars represent standard deviation. Nos. 1–10 indicate the seedlings that were inoculated with serially diluted bacterial suspensions: 3.1×10^8^, 6.25×10^7^, 1.25×10^7^, 2.5×10^6^, 5.0×10^5^, 1.0×10^5^, 2.0×10^4^, 4.0×10^3^, 750∼800 and 150∼200 CFU ml−1. (TIF) [file pone.0051410.s007.tif]

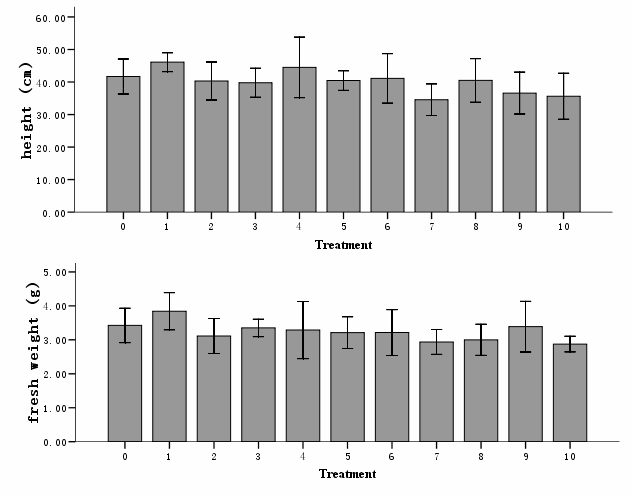

Supplement: Figure S8 — The heights and fresh weights of A. annua shoots grown for 74 days after YIM 63111 inoculation compared with untreated plants (No. 0). Nos. 1–10 indicate the seedlings that were inoculated with serially diluted bacterial suspensions: 1.84×10^9^, 3.68×10^8^, 7.38×10^7^, 1.48×10^7^, 2.96×10^6^, 5.90×10^5^, 1.18×10^5^, 2.36×10^4^, 4.72×10^3^ and 900∼1.0×10^3^ CFU ml−1. Error bars represent standard deviation. (TIF) [file pone.0051410.s008.tif]
