# Supplementary material for: An Endophytic Pseudonocardia Species Induces the Production of Artemisinin in Artemisia annua
Source: PLoS One. 2012 Dec 12;7(12):e51410. doi: 10.1371/journal.pone.0051410 (PMC3520919; doi:10.1371/journal.pone.0051410)
Supplement: Table S1 — Different characteristics of strain YIM 63111 and its closely related Pseudonocardia species. (DOC) [file pone.0051410.s009.doc]

**Table S1** **Different characteristics of strain YIM 63111 and its closely related *Pseudonocardia* species.**

| **Characteristic** | **1** | **2** | **3** | **4** |
| --- | --- | --- | --- | --- |
| Utilization of: |  |  |  |  |
| Dulcitol | - | + | + | - |
| Glycerol | + | + | + | - |
| *myo*-Inositol | - | + | + | + |
| D-Mannitol | + | w | + | + |
| D-raffinose | + | + | + | - |
| D-Ribose | + | + | w | + |
| D-sorbitol | - | + | + | + |
| Hydrolysis of: |  |  |  |  |
| Gelatin | - | + | + | - |
| Starch | - | + | + | -  + |
| Reduction of nitrate | - | + | + | - |
| H_2_S production | + | - | - | - |
| Growth at/on: |  |  |  |  |
| 10 ºC | + | + | + | - |
| 45 ºC | + | + | - | + |
| 5 % NaCl | + | + | + | - |

Strains: 1, strain YIM 63111; 2, *Pseudonocardia alni* DSM 44104^T^; 3, *Pseudonocardia antarctica* DSM 44749^T^; 4, *Pseudonocardia carboxydivorans* JCM 14827^T^

+, Positive; w, weakly positive; -, negative.
